# Preparation of an Amidated Graphene Oxide/Sulfonated Poly Ether Ether Ketone (AGO/SPEEK) Modified Atmosphere Packaging for the Storage of Cherry Tomatoes

**DOI:** 10.3390/foods10030552

**Published:** 2021-03-07

**Authors:** Yao Cheng, Hao Dong, Yuanyue Wu, Kaijun Xiao

**Affiliations:** 1 School of Food Science and Technology, South China University of Technology, 381, Wushan Rd., Tianhe District, Guangzhou 510641, China; yaoyao950920@163.com (Y.C.); wuyuanyue213@163.com (Y.W.); 2 School of Food Science and Technology, Zhongkai University of Agriculture and Engineering, 24, Dongsha Street, Fangzhi Rd., Haizhu District, Guangzhou 510225, China; donghao@zhku.edu.cn

**Keywords:** amidated graphene oxide, sulfonated poly ether ether ketone, modified atmosphere film, cherry tomatoes, food packaging

## Abstract

The shelf life of cherry tomatoes is short so that new and efficient preservation techniques or procedures are required to reduce postharvest losses. This study focused on the development of a sulfonated poly ether ether ketone (SPEEK) film incorporated with amidated graphene oxide (AGO), for the storage of cherry tomatoes in modified atmosphere packaging. The mechanical properties, gas permeability, and moisture permeability were subsequently tested. The evolution of attributes related to shelf life, such as gas composition, physicochemical properties, and sensory properties were also monitored during storage trials. AGO, as an inorganic filler, increases the thermal stability and mechanical properties of SPEEK-based films, while it reduces the water absorption, swelling rate, and moisture permeability. Importantly, all the AGO/SPEEK films exhibited enhanced gas permeability and selective permeability of CO_2_/O_2_ relative to the SPEEK film. Moreover, 0.9% (*w/w*) AGO/SPEEK film showed an enhanced permeability coefficient of CO_2_, corresponding to an increase of 50.7%. It could further improve the selective coefficient of CO_2_/O_2_ to 67.1%. The results of preservation at 8 °C revealed that: 0.9% (*w/w*) AGO/SPEEK film was significantly effective at maintaining the quality and extending the shelf life of cherry tomatoes from 15 to 30 days, thereby suggesting the potential for applying AGO-incorporated SPEEK films for food packaging materials.

## 1. Introduction

Cherry tomatoes (*Lycopersicon esculentum var. cerasiforme cv.*) consist of beneficial nutrients, such as minerals, vitamins, phenolic compounds, and lycopene [1]. However, cherry tomatoes deteriorate rapidly without proper preservation, and thus their shelf life ranges from only 5 to 7 days after harvest [2]. Therefore, it is very important to reduce decay incidence and extend the postharvest life of cherry tomatoes. Currently, different treatments, such as drying, edible coatings, modified atmosphere packaging, etc., have been applied to maintain the postharvest quality of cherry tomatoes [2,3,4]. However, modern consumers’ convenient lifestyle and their demand for minimally processed fruits and vegetables put forward higher requirements for freshness preservation [4]. Modified atmosphere packaging, as one of the most widely used techniques for fresh produce preservation, has attracted significant research interest for its time-saving convenience and ease of use. It reduces gas exchange, moisture evaporation, and respiration reaction rates and suppresses physiological disorders of fruits and vegetables [5]. Existing modified atmosphere packaging usually involves the use of polymer materials, such as polyethylene (PE), polyvinyl chloride (PVC), and polypropylene (PP) [4]. However, these films have limited gas selectivity or gas permeability, which can no longer meet the requirements for the preservation of cherry tomatoes [6]. From this perspective, it is of great practical significance to develop an atmosphere-modified packaging that matches the respiration rate of cherry tomatoes [7].

Among diverse polymers, sulfonated poly ether ether ketone (SPEEK), as an aromatic thermoplastic engineering plastic, has attracted increasing attention for the development of food packaging materials due to its excellent gas permeability [8]. For instance, He et al. [9] prepared a SPEEK/PVDF self-balancing packaging film, which significantly shortened the equilibrium time of CO_2_ or O_2_ concentration in the package, making the shelf life of broccoli more than double. However, there is still much room to improve the selective permeability of SPEEK. Noteworthily, these polymers can be further improved as functional packaging materials by incorporating some new compounds, such as nanoparticles [10]. Graphene oxide (GO), as a newly emerging carbon nanomaterial with a two-dimensional layered structure, has a large specific surface area and strong CO_2_ adsorption capacity, and thus acts as one of the ideal materials [11,12]. At present, the methods for the surface modification of GO can be categorized as covalent and non-covalent functionalization [13]. Among them, the amidated form of GO (AGO) better improves the adsorption of CO_2_ and effectively prevents the deterioration of mechanical properties of the film. However, the effects of AGO/SPEEK films on postharvest attributes and flavor characteristics in cherry tomatoes have not been reported yet.

In the present study, GO was modified by amidation and then added to SPEEK in the form of inorganic fillers to prepare a film with a high CO_2_/O_2_ separation ratio. The produced films were then characterized to analyze the efficacy of modification of SPEEK with AGO. Subsequently, the mechanical properties, water uptake ratio (WUR), swelling ability (SA), water vapor permeability (WVP), and gas permeability of the pure SPEEK film and AGO/SPEEK films were also examined and compared. Finally, the efficacy of the resulting package was assessed by performing storage trials of cherry tomatoes.

## 2. Materials and Methods

### 2.1. Materials and Chemicals

Cherry tomatoes were obtained from a local farm in Guangdong province, China. PP boxes (1 L) were obtained from a local store (Walmart, Guangzhou, China), with no obvious visual defects. GO was obtained from Sigma-Aldrich Corp. (Shanghai, China). PEEK (Victrex™ 450 G) was obtained from Victrex Co., Ltd. (Victrex, UK). CO_2_ (99.99%), O_2_ (99.99%), and N_2_ (99.99%) were obtained from Air Chemical Co., Ltd. (Guangzhou, China). All other analytical chemical reagents were achieved from Aladdin Industrial Corporation (Shanghai, China) and used as received.

### 2.2. Synthesis of AGO

GO powder (~200 mg) was dispersed in *N*,*N*-dimethylformamide (DMF) by ultrasonication. The mixture was mechanically stirred at 30 °C for 1 h and then slowly dropped into ethylenediamine (EDA, 300 mL) under rapid stirring. The reaction was allowed to proceed for 24–48 h under nitrogen protection. After the reaction, the mixture was diluted in ethanol (1600 mL) and washed several times to ensure the removal of excess EDA. Finally, AGO could be collected after filtration and dried overnight in a vacuum oven at 50 °C [13,14,15].

### 2.3. Preparation of AGO/SPEEK Films

SPEEK (DS 79.8%) and AGO/SPEEK films were prepared by the method proposed by He et al. [9,12,15]. SPEEK was dissolved in DMF to form a 10% (*w/w*) mixed solution under magnetic stirring. Then, AGO (0.1%, 0.5%, and 0.9%) was added to react with SPEEK for 24 h at 60 °C in order to obtain a casting solution. The casting solution was defoamed at room temperature under vacuum. Subsequently, it was cast on a clean glass plate, and its thickness was controlled at 40 μm using an adjustable scraper. The casted glass plate was placed horizontally into a vacuum oven at 60 °C and dried for 24 h to completely remove the solvent. Finally, the dried film was peeled off from the glass plate for further tests.

### 2.4. Characterization

The structure and functional groups of the surface of the films were analyzed by Fourier-transform infrared spectroscopy (FTIR, 2000 GX spectrometer, Perkin Elmer, Waltham, MA, USA) at 4000 to 400 cm^−1^. The structure of the molecules was determined using a Renishaw micro-Raman spectroscopy system with a laser wavelength of 532 nm (Raman, RENISHAW PLC, Kingswood, UK). Crystalline properties were evaluated by X-ray diffraction (XRD, D8 Advance X-ray diffractometer, Bruker, Germany). The morphology and microstructure of the films were inspected by scanning electron microscopy (SEM, SU-3500, Hitachi, Japan) at an accelerating voltage of 10 kV and by transmission electron microscopy (TEM, JEM-2100F, Tokyo, Japan). The thermal properties of the films were determined from 25 to 800 °C at a heating rate of 10 °C min^−1^ in a nitrogen atmosphere by thermogravimetric analysis (TGA, Metter-Toledo, Switzerland). The composition and chemical state of the elements in the films were determined by X-ray photoelectron spectroscopy (XPS, K-Alpha^+^ spectrometer, Thermo-Fisher, Waltham, MA, USA). 

### 2.5. Film Properties

#### 2.5.1. Mechanical Properties

Mechanical properties, including elongation at break (EAB), tensile strength (TS), and elastic modulus (EM), were determined by tensile testing, using an LR 5K universal testing machine (Lloyd Instrument, Bognor Regis, UK). The film was cut into rectangular strips with dimensions of 60 mm × 20 mm. The deformation was recorded at a crosshead speed of 10 mm min^−1^ and an initial gage length of 50 mm. Averages were calculated from five replicates of each sample.

#### 2.5.2. Swelling Ability

Film swelling was determined by following the method proposed by Banerjee et al. [16]. A film (30 mm × 30 mm) was dried at 100 °C for 2 h and immersed in distilled water at room temperature for 24 h. The WUR and area swelling ratio (ASR) of the film were calculated by the weight difference of the film before and after water absorption.

#### 2.5.3. Water Vapor Permeability 

The films were cut into pieces with size of 4 cm × 4 cm. The water vapor permeability of the film was measured following the method provided by Shima Jafarzadeh [17]. The water vapor transmission rate (WVTR) was measured from the slopes (linear) of the steady-state portion of weight loss of the cup versus time curve. Further, the WVP of the films was measured as follows:

(1) $$\mathrm{WVP}=\mathrm{WVTR}\times \frac{D}{\Delta p}$$

where D is the thickness of the film and ∆p is the partial water vapor pressure difference over the two sides of the film.

#### 2.5.4. Gas Permeability

The theory of measuring the gas permeability of the film uses the traditional differential pressure method. The films were cut into a 10 cm × 10 cm size and were measured with a BSV-1A gas permeability tester (Lab Stone, Guangzhou, China). The single gas permeability of the film was determined by changing the gas source (O_2_, CO_2_, or N_2_) in the room temperature. The gas permeability coefficient of the film (P) is calculated as follows:

(2) $$P=\frac{\Delta p}{\Delta t}\times \frac{V}{S}\times \frac{T_{0}}{P_{0}\times T}\times \frac{D}{\left(p_{1}-p_{2}\right)}$$

The ideal gas selectivity of the film can be obtained by calculating the quotient of the permeability of the film under different gas sources.

Where ∆p/∆t is the arithmetic mean of changes in hourly gas pressure in the infiltration chamber; V is the volume of the infiltration chamber; S is the area of the film; T is the test temperature; p1−p2 is the pressure difference between the two sides of the film; T_0_ and p_0_ are the temperature and pressure under standard conditions, which are 273.15 K and 101,315 Pa, respectively, and D is the thickness of the film.

### 2.6. Pretreatment and Storage of Cherry Tomatoes

Fresh cherry tomatoes without mechanical damage were manually screened and precooled at 4 ± 1 °C and 90% relative humidity for 24 h. The samples (250 ± 10 g) were randomly packed in a 1 L packaging box (PP), respectively. The surface of the box was sealed with AGO/SPEEK films using a JSMAP-1D100F-S semi-automatic modified atmosphere packaging machine (JuGang Machinery Manufacturing Co., Ltd., Shanghai, China). The temperature, pressure, and heat-sealing time of the packaging machine were 110 ± 5 °C, 1.8 kPa, and 1 s, respectively. Samples without packaging were set as control group. The packaged samples were stored in a thermo-hygrostat at 8 ± 1 °C for 30 days. The physiological and biochemical properties of cherry tomatoes were evaluated every 5 days.

#### 2.6.1. Internal Atmosphere Composition 

Concentrations of CO_2_ and O_2_ in the box were monitored using an OXYBABY M+ gas analyzer (Zillion Corporation, Shanghai, China). During the measurement, a customized sealing gasket with a size of 1 cm × 1 cm was pasted on the package, and then the needle of the gas analyzer was inserted into the package. The sensor of the gas analyzer that connected to the needle automatically collects gas samples for measurement in 10 s and the actual values of O_2_ and CO_2_ volume ratio (%) would be displayed on the screen of the gas analyzer in real time.

#### 2.6.2. Sensory Properties

In the sensory evaluation experiment, 10 professional sensory evaluators conducted the experiment on color, odor, texture, and other indicators of cherry tomatoes according to sensory scoring standards. A 5-point scale (0, unacceptable to 5, excellent) was used. In the sensory laboratory, under a cool white fluorescent lamp, numbered samples were randomly placed in small dishes and provided to each sensory evaluator [9].

#### 2.6.3. Physicochemical Properties

The hardness of cherry tomatoes was measured using a GY 2 handheld hardness meter (Minks Testing Equipment Co., Ltd., Xi’an, China). Hardness values were determined as maximum force registered in the force vs. time curves. Measurements were made on five fruits per experimental unit.

Color parameters (L*, a*, b*) of cherry tomatoes were measured using a CR20 colorimeter (Konica Minolta Sensing Inc, Tokyo, Japan). For each experimental unit, 10 tomatoes were measured and the results are presented as ΔE [18].

Cherry tomatoes (125 g) were homogenized with distilled water (125 mL) for 5 min using a high shear mixing homogenizer, and the pH of the mixture was determined using an FE20 digital pH meter (Mettler Toledo, Zurich, Switzerland). 

The weight of cherry tomatoes was recorded at the initial day and sampling day by using an AUY220 analytical balance (Shimadzu Corporation, Kyoto, Japan). The weight loss (%) was calculated following the method described by Araguez et al. [19].

The total soluble solid (TSS) content of cherry tomatoes was determined using an AR 2008 Abbe refractometer (Kruess, Hamburg, Germany). A drop of clear tomato juice obtained by squeezing the fruit in a muslin cloth was placed in the reading chamber, and the value of TSS was read (%) [20]. 

The content of vitamin C (V_C_) was determined using a UV1601 spectrophotometer (Rayleigh Analytical Instruments Co., Ltd., Beijing, China) following the method reported by Hou et al. [21]. The V_C_ content was calculated and expressed as mg per 100 g of sample based on weight.

### 2.7. Statistical Analysis

Statistical analyses were performed by using the SPSS software (SPSS Inc., Chicago, IL, USA). All experiments were conducted in triplicates and all data presented as mean ± standard deviation were subjected to analysis of variance (ANOVA). Duncan’s multiple range tests were used to compare the difference among mean values at a level of 0.05.

## 3. Results and Discussion

### 3.1. Analysis of AGO

**Figure 1:**
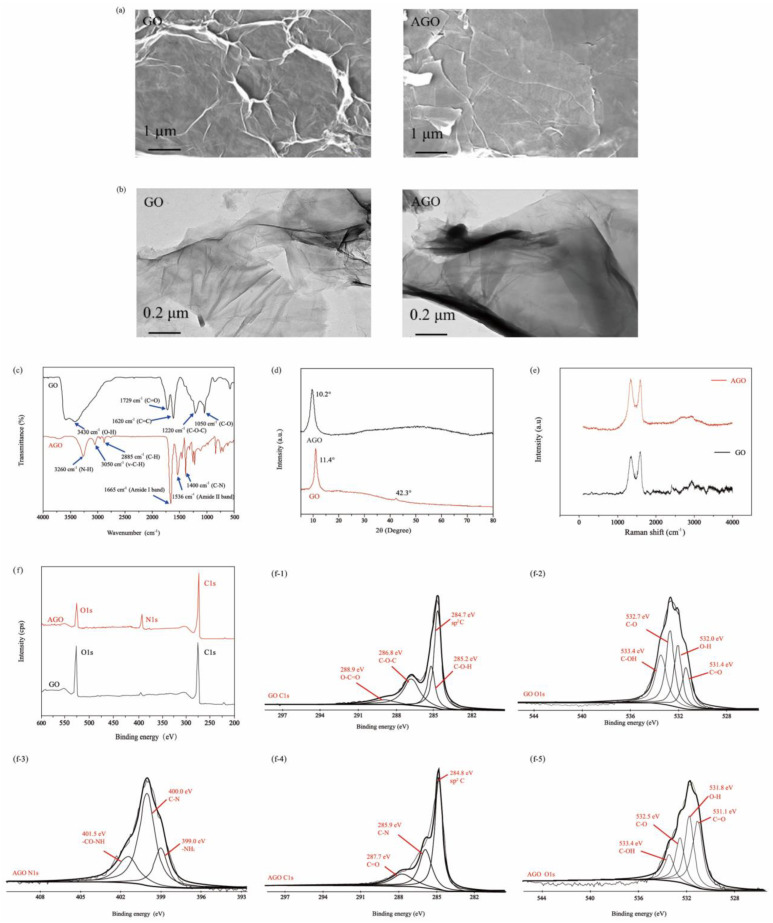
Characterization of amidated graphene oxide (AGO). (**a**) SEM, (**b**) TEM, (**c**) FTIR spectra, (**d**) XRD pattern, (**e**) Raman spectra, (**f**) XPS survey spectra, (**f-1**) C1s spectra of GO, (**f-2**) O1s spectra of GO, (**f-3**) N1s spectra of AGO, (**f-4**) C1s spectra of AGO, and (**f-5**) O1s spectra of AGO.

SEM was performed to characterize the microscopic morphology of AGO. Figure 1a shows that AGO was stacked together to form a multilayer sheet structure, which is consistent with the reported of Ramanathan et al. [20,21,22]. Compared with GO, Figure 1b shows TEM images, further showing a transparent yarn-like structure with fewer wrinkles and smoother surface of AGO [15,20]. It was attributed to the partial reduction of GO by EDA and the interaction between the AGO plates, which together led to decrimping and blurring of the edges [14,15,23].

The FTIR spectra of AGO are shown in Figure 1c. The obvious disappearance of stretching peaks of O−H (3430 cm^−1^) and C=O (1729 cm^−1^), and the appearance of stretching peaks of N−H (3260 cm^−1^) and C−H (3050, 2885 cm^−1^) both indicate the partial modification of GO by EDA [23,24]. Moreover, for AGO, three new peaks appear at 1665, 1536, and 1440 cm^−1^, which are assigned to the C=O stretching of the amide I band and the combined absorption caused by N−H bending and C−N stretching in the amide II band, respectively. It is attributed to the amidation reaction or the substitution reaction between EDA and GO, which is consistent with the XRD results (Figure 1d).

The Raman spectra of GO and AGO are shown in Figure 1e. A Raman D-band and a G-band of GO are observed at 1354 and 1600 cm^−1^, corresponding to the structure defects on the graphene sheets and the sp^2^ hybridization of the hexagonal carbon structure, respectively. Compared to the Raman spectra of GO, the D-band and G-band of the AGO shift to 1340 and 1595 cm^−1^, respectively. In general, the relative intensity of the I_D_/I_G_ ratio partly indicates the quality of grapheme [25]. Herein, the I_D_/I_G_ ratio of GO (1.41) was lower than that of AGO (1.60), indicating a slight reduction of GO, which was attributed to the conversion of carbon atoms from the sp^3^ to the sp^2^ state [26]. 

**Figure 2:**
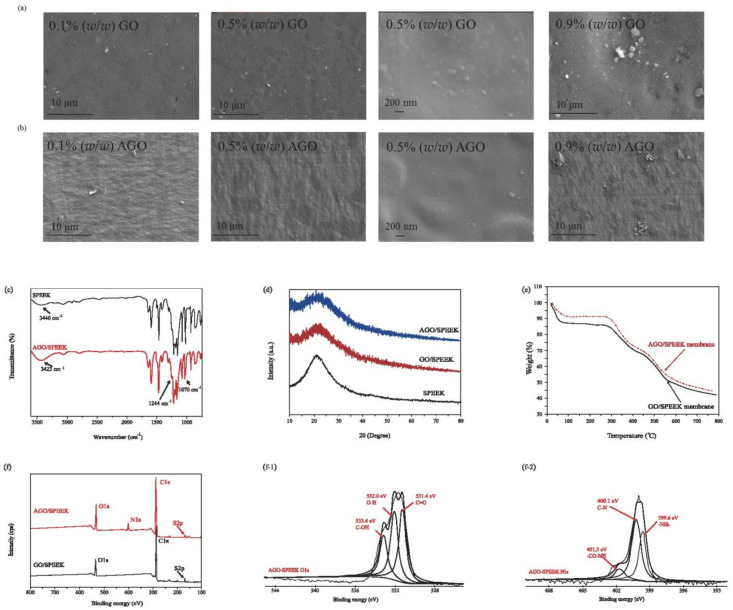
Characterization of films. (**a**) SEM of GO/SPEEK films, (**b**) SEM of AGO/SPEEK films, (**c**) FTIR spectra, (**d**) XRD pattern, (**e**) TGA curves, (**f**) XPS survey spectra, (**f-1**) O1s spectra and (**f-2**) N1s spectra of 0.5% (*w/w*) AGO/SPEEK films.

**Figure 3:**
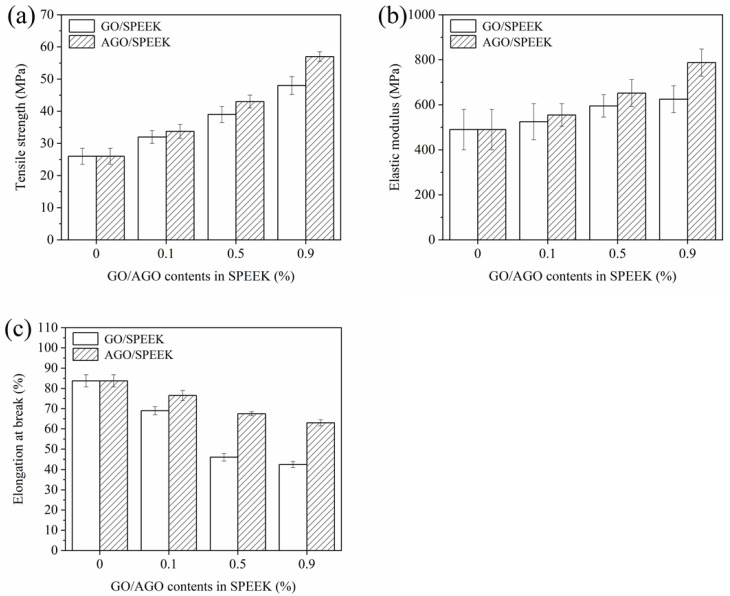
Mechanical properties of AGO/SPEEK films. (**a**) Tensile strength, (**b**) elastic modulus, (**c**) elongation at break.

**Figure 4:**
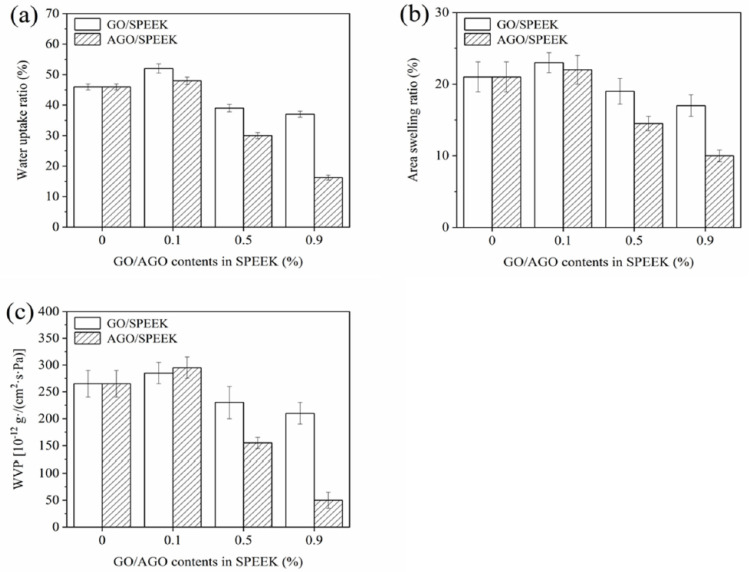
Water permeability of AGO/SPEEK films. (**a**) Water uptake ratio, (**b**) area swelling rate, (**c**) water vapor permeability.

**Figure 5:**
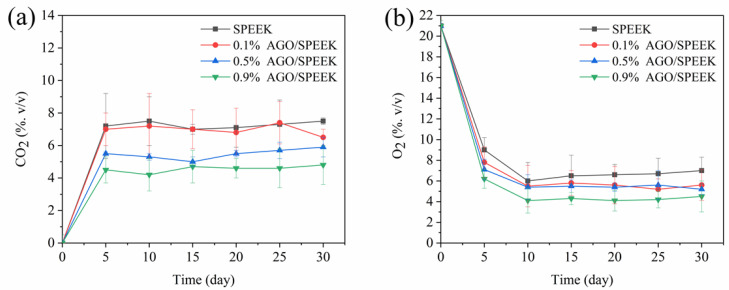
Internal atmosphere composition under AGO/SPEEK films packaging in the storage of cherry tomatoes. (**a**) CO_2_, (**b**) O_2_.

The composition and chemical state of elements of AGO were further researched by XPS. Figure 1f, demonstrates that the full spectrum of AGO shows a significant decrease in O1s and an increase in C1s, accompanied by the generation of a new peak N1s, indicating the successful modification of GO. Through further analysis, it was found that the changes in the intensity of O1s component in Figure 1f were attributed to the disappearance or the decrease of the intensity of the C−OH group (285.2 eV), C–O–;C group (286.8 eV), and O=C–O (288.9 eV) group in Figure 1f-1. The new peak at the binding energy of 285.9 eV of C1s component in Figure 1f-4 corresponded to the C–N group, which was proved by the three main Gaussian peaks at binding energies of 399.0, 400.0, and 401.5 eV of N1s component in Figure 1f-3, assigned to –NH_2_, C–N, and –CO–NH groups, respectively. Furthermore, the O1 spectrum of AGO shown in Figure 1f-5 also indicates the decrease in the strength of the oxygen-containing groups to varying degrees, which further reveals that the amidation reaction between EDA and GO is mainly caused by amino groups and oxygen-containing groups [23,27].

### 3.2. Characterization Analysis of AGO/SPEEK Films

For any blending system, an important effect that should be addressed is the miscibility among different compounds as it determines structural stability and physicochemical properties of the blend. In this study, good miscibility between SPEEK and AGO resulted in the formation of transparent and homogeneous blending solutions and films. More obvious details were obtained based on the microstructure of the material. As shown in Figure 2a,b, there was a partial depressions on the GO/SPEEK films with no sheet structure, which is similar to the result of Dai et al. [12,28,29]. The surface of AGO/SPEEK films displayed a relatively rough morphology with a more obvious layered structure compared to GO/SPEEK films, consistent with the typical morphology of AGO [12,23]. Notably, most of the AGO was evenly dispersed in the SPEEK matrix, while small-scale accumulation gradually appeared with an increase in the AGO loading, indicating that the two compounds were homogeneous [28].

Numerous oxygen-containing functional groups, such as carbonyl (C=O), hydroxyl (–OH), carboxyl (–COOH), as well as amino groups, are present on both sides of AGO; therefore, the interfacial interactions between AGO and SPEEK matrices should be verified. Figure 2c shows the FTIR spectra of AGO/SPEEK films. The AGO/SPEEK film shows a characteristic broad peak corresponding to intermolecular hydrogen bonds, which is reflected in the shift of the −OH group band from 3440 to 3423 cm^−1^. The bands at 1080 and 1250 cm^−1^ are associated with asymmetrical and symmetrical stretching vibrations of the O=S=O group, while for AGO/SPEEK film, the corresponding bands are shifted to 1070 and 1244 cm^−1^. The composition and chemical state of elements of AGO/SPEEK films were also researched by XPS (Figure 2f), which was found to be similar to the XPS results of AGO and revealed the blending mechanism between AGO and SPEEK. Figure 2d shows XRD results, clearly indicating the formation of hydrogen bonds between the sulfonated acid groups in SPEEK and polar or amino groups on AGO. Furthermore, TGA (Figure 2e) confirmed that the interface interaction caused by hydrogen bonds between AGO and SPEEK matrix was stronger, leading to the shift in the TG curve toward high temperature, indicating the improvement of thermal stability [12,29].

### 3.3. Analysis of Film Properties

#### 3.3.1. Mechanical Properties

The mechanical properties of films represent their ability to maintain their integrity and endure external stress during the processing, transportation, handling, and storage of packaged materials [30]. As shown in Figure 3, AGO/SPEEK film presented the highest TS (Figure 3a) and EM (Figure 3b) but the lowest EAB (Figure 3c) under the maximum load of 0.9% (*w/w*). It was attributed to the existence of numerous hydrogen bonds in the AGO/SPEEK film that caused strong cohesive energy density [12,31]. Likewise, AGO/SPEEK films also presented higher TS and EM under the same load, which was ascribed to the reaction of more –NH_2_ groups with SPEEK as indicated by the decrease of crystallinity shown in XRD pattern [29]. The EAB of AGO/SPEEK films (Figure 3c) was lower than that of the original film by varying degrees, which was imputed to the loading of AGO that restricted the movement of SPEEK polymer chains [32]. Moreover, extensive hydrogen bonds increased the interfacial adhesion, thereby enhancing the mechanical properties of the film [12,31]. Noteworthily, when the filling amount of the inorganic filler was between 0.1% and 0.9% (*w/w*), the EAB of films remained above 40%, showing that it still had sufficient mechanical strength and extensibility, which is required for food packaging applications [30].

#### 3.3.2. Swelling Ability

The swelling ability is an important factor in composite films and represents its water absorption resistance property and type of film use. The water absorption tendency of the film affects the swelling ability to a certain extent. Consequently, the water absorption of the film shows a trend similar to that of the swelling ability. As shown in Figure 4a,b, the swelling ability of both the films shows a trend that first increases and then decreases, and this was mainly attributed to the influence of functional groups. When the added amount of the inorganic filler (GO/AGO) was less than 0.1% (*w/w*), the hydrophilic functional groups in the inorganic filler resulted in temporary increase in the swelling ability of the film. However, when the amount was more than 0.1% (*w/w*), “blocking effect” caused by the increase of functional groups played the main role, leading to a decline in swelling [15,31]. Specifically, the area swelling ratio of films with 0.5% and 0.9% (*w/w*) AGO was significantly reduced, reaching 15% and 10%, respectively. Moreover, the area swelling ratio of the AGO/SPEEK film was lower than that of the GO/SPEEK film under the same loading, which was ascribed to the less hydrophilic amide groups introduced by AGO, and it narrowed the gap of the SPEEK polymer chain. Thereby, it can be concluded that the incorporation of AGO and SPEEK matrix can improve the usability of films for high-humidity food packaging.

#### 3.3.3. Water Vapor Permeability

WVP is one of the most important functional properties for a food packaging film [30]. Film with low WVP has a good water vapor barrier property, which can effectively reduce moisture transfer between the surrounding atmosphere and the food environment. As shown in Figure 4c, the SPEEK film has relatively high moisture permeability, which is attributed to the presence of abundant sulfonic acid groups that gather together to form hydrophilic domains that generate hydrophilic transport channels [33]. The WVP result of AGO/SPEEK films was found to be similar to the results of WUR. One reason was that dispersed AGO constituted a barrier and hindered the migration of water molecules through the film, while the other was that AGO could extend the path of water molecules through the film, thereby delaying the time to penetrate the film [31]. However, it can be seen that the WVPs of AGO/SPEEK films were both above 50 × 10^−12^ g·cm/(cm^2^·s·Pa), which is higher than that of PE [34]. Therefore, the AGO/SPEEK films were found to be suitable for the preservation of fruits with high moisture content.

#### 3.3.4. Gas Permeability

Blending changes the porosity of film and thus also the gas permeability for different gases [9]. The pure gas permeability and ideal selectivity of AGO/SPEEK films are summarized in Table 1. SPEEK has superior gas permeability performance because of the presence of a polar –SO_3_H group chain, which interacts with different gas molecules and then improves upon the solubility coefficients with the gas particles [9,35]. Comparative analysis indicates that the gas permeability of GO/SPEEK films decreases slightly due to the consumed –SO_3_H groups, which is consistent with the results of Xin et al. [36]. Notably, the CO_2_ permeability of AGO/SPEEK films was significantly increased, while the permeability of O_2_ or N_2_ did not change significantly. It was attributed to the presence of –NH_2_ groups in AGO that have an excellent affinity for CO_2_. Moreover, the reaction between CO_2_ and –NH_2_ groups intensified in the wet state, resulting in increased CO_2_ permeability of AGO/SPEEK films [34]. Therefore, for both CO_2_/O_2_ and CO_2_/N_2_, ideal selectivity increased from 7.3 and 22.2 to 12.2 and 29.1, respectively, with increasing AGO composition in the blended films.

### 3.4. Quality Analysis of Cherry Tomatoes

#### 3.4.1. Internal Atmosphere Composition

The gas permeability of the film is an important factor affecting the exchange of internal and external gases. The respiration of fruits and vegetable produces CO_2_ and consumes O_2_ in the microenvironment of package. On account of relative gas pressure differences between the inside and outside of the package, CO_2_ gets released to the atmosphere and O_2_ will enter the package. Thus, films with appropriate gas permeability to O_2_ and CO_2_ can significantly prolong the shelf life of fruit and vegetables. As shown in Figure 5a, the CO_2_ concentration inside the package maintained an equilibrium concentration of 4–7%, while the corresponding O_2_ concentration was 4–6% (Figure 5b). The difference in gas concentration in the final package was not obvious, although the loading of AGO was different, which may be caused by the originally low loading of AGO. However, the higher the loading of AGO, the lower the final equilibrium concentration of CO_2_ in the package. Accordingly, it was speculated herein that the modified atmosphere packaging with an AGO loading of 0.5–0.9% (*w/w*) is more suitable for preservation.

#### 3.4.2. Sensory Properties

**Figure 6:**
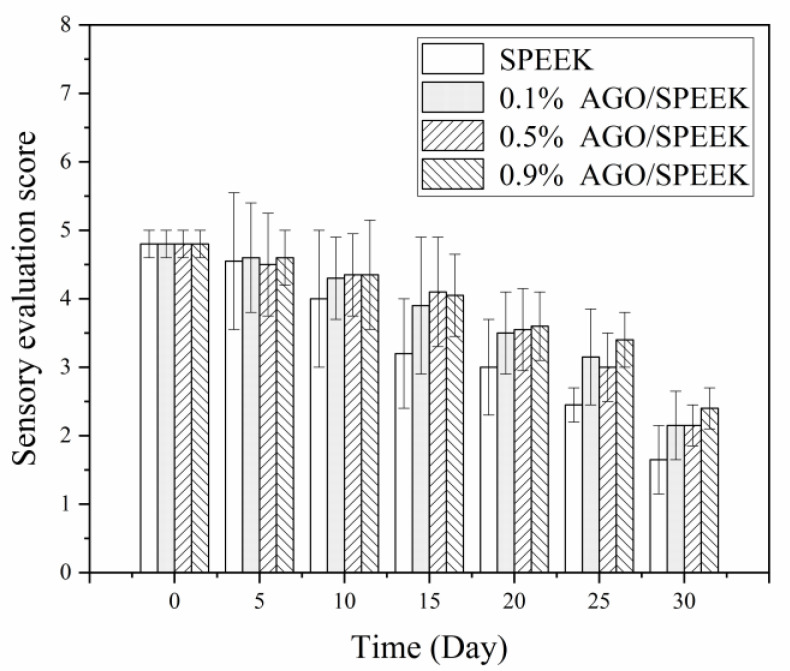
Sensory evaluation scores of AGO/SPEEK films in the storage of cherry tomatoes (a 5-point scale: 1 means unacceptable, 2 means barely accepted, 3 means general, 4 means good, and 5 means excellent).

The sensory properties such as color, hardness, and overall acceptability of cherry tomatoes packaged with AGO/SPEEK films were determined during storage, and their results are presented in Figure 6. In general, cherry tomatoes were ranked almost similar with respect to their sensory properties; however, packaged cherry tomatoes had better peel color when the AGO content in the package was higher. Interestingly, cherry tomatoes packaged with 0.9% (*w/w*) AGO/SPEEK films maintained acceptable sensory scores even after 30 days of storage, showing the potential of AGO/SPEEK film in extending the shelf life.

#### 3.4.3. Physicochemical Properties

**Figure 7:**
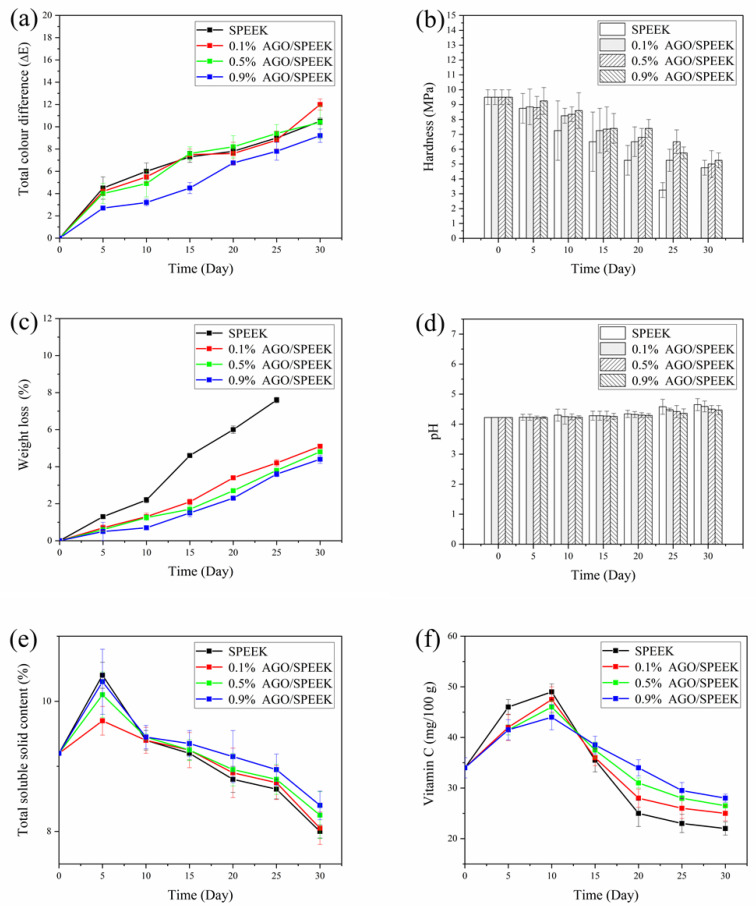
Physicochemical properties of AGO/SPEEK films in the storage of cherry tomatoes. (**a**) Total color difference, (**b**) hardness, (**c**) weight loss, (**d**) pH, (**e**) total soluble solid content, (**f**) vitamin C.

Changes in color, hardness, weight loss, pH, TSS content, and Vc content of cherry tomatoes packaged with AGO/SPEEK films during storage are shown in Figure 7. Color is the main external quality criteria affecting the marketability [4]. Figure 7a demonstrates that the ΔE of cherry tomatoes in different packages shows an increasing trend, and color changes of AGO/SPEEK films packaged are relatively minimal in different periods, probably owing to the reduction of ethylene synthesis influenced by the high content of CO_2_. This is in good agreement with the gas permeability results of AGO/SPEEK films discussed earlier. Specifically, high loading of AGO leads to enhanced CO_2_/O_2_ selective permeability of the film and it is easier to form a high CO_2_ and low O_2_ gas atmosphere, which thus slows down the accumulation of pigment and the browning process of tissues caused by the aging of cherry tomatoes [4,21].

Furthermore, hardness of cherry tomatoes is an important factor for the estimation of maturity [37]. Figure 7b illustrates that the hardness of cherry tomatoes in all groups exhibited a decreasing trend with the extension of storage time, and the control group was more significant, which depends on the progress of the conversion of insoluble pectin to soluble pectin or even pectin acid [38]. The hardness of cherry tomatoes packaged with 0.9% (*w/w*) AGO/SPEEK film decreased by 47.4%, which is similar to the result reported by Zhang et al. [37]. Thus, the AGO/SPEEK film can efficiently slow down the softening of the cherry tomatoes.

Transpiration and respiration of the fruits during storage lead to loss of water and weight, which can cause a wilting phenomenon and shorten the postharvest life of fresh fruit. According to the reported, cherry tomatoes will cause severe wilting when the weight loss is about 11%, while at about 6% weight loss they will not wilt [39,40]. Figure 7c shows that the weight loss of cherry tomatoes was the least when the packaging was done with 0.9% (*w/w*) AGO/SPEEK film and exhibited an increasing trend in the following order: 0.5% (*w/w*), 0.1% (*w/w*), and the original film. Undoubtedly, the samples in the control group were significantly softened and cracked and exhibited the highest loss of weight of up to 8% after 30 days of storage, while that of others increased to 3–4%, which benefits from the water resistance of films inhibiting the evaporation of water. Notably, that is coherence with the results of WVP shown in Figure 4c.

Moreover, pH is an important reference for evaluating the quality of cherry tomatoes during storage because it uses organic acids as a substrate for respiration [19]. Figure 7d shows a slight increasing trend of pH from 4.2 to 4.8, which coincided with the results of hardness caused by increasing pectin acid. The pH of control samples increased more significantly mainly resulting from no packaging, which is consistent with the report by D’Aquino et al. [41].

TSS refers mainly to soluble sugars and is a very important indicator for determining consumer acceptability [42]. Figure 7e exhibits that the TSS content increased at the beginning of storage (5 days), benefiting from the accumulation of nutrients brought by maturity. However, it decreased to varying degrees in the later stage, caused by the hydrolysis of sucrose for respiration and maintaining the physiological activity of plant raw materials [42]. Noteworthily, cherry tomatoes packaged in a 0.9% (*w/w*) AGO/SPEEK film showed a slower decrease in TSS compared with others, indicating a delay in the ripening of cherry tomatoes under high concentration of CO_2_ [43].

Figure 7f shows that the Vc content in cherry tomatoes increased initially and then decreased during the storage period, similar to the behavior described by Zhou et al. [30]. The increased V_C_ content of cherry tomatoes was attributed to the accumulation of post-ripening effect, while the decrease resulted from the high rates of respiration, oxidative deterioration, and accumulation of CO_2_ [30,41]. Noteworthily, the V_C_ content of cherry tomatoes packaged in 0.5% (*w/w*) or 0.9% (*w/w*) AGO/SPEEK films was basically maintained at 30 mg/100 g at the end of storage, much higher than others, benefited from favorable oxygen barrier properties of AGO/SPEEK films that effectively prevented the oxidation of V_C_.

## 4. Conclusions

AGO/SPEEK films were prepared by physical blending for modified atmosphere packaging. A series of data revealed that the loading of AGO improves the mechanical properties, gas permeability, and selective permeability of CO_2_/O_2_ compared to the original film, while reducing the water uptake ratio, the swelling ability, and the water vapor permeability. Preservation experiments revealed that 0.9% (*w/w*) AGO/SPEEK films delayed the maturation process and doubled the shelf life of cherry tomatoes. Overall, these findings demonstrated that AGO/SPEEK films have great application potential in the preservation of fruits with high moisture content and low respiration rate. Undeniably, many more systematic explorations are still demanded to confirm the safety and service life of the film when storing various food products, which will be pursued in the future.

## Figures and Tables

**Table 1:** Gas permeability of films for pure gas.

SPEEK Films	Permeability (Barrer)			Ideal Gas Selectivity (α)	
	CO_2_	O_2_	N_2_	CO_2_/O_2_	CO_2_/N_2_
0	14.68	2.02	0.66	7.3	22.2
0.1% (*w/w*) GO	13.26	1.96	0.60	6.7	22.1
0.5% (*w/w*) GO	12.31	1.80	0.65	6.8	18.9
0.9% (*w/w*) GO	13.92	1.85	0.62	7.5	22.4
0.1% (*w/w*) AGO	16.81	2.06	0.66	8.1	25.5
0.5% (*w/w*) AGO	19.40	1.84	0.68	10.5	28.5
0.9% (*w/w*) AGO	22.12	1.82	0.76	12.2	29.1

## Data Availability

The study did not report any data.
